# Delayed Time-to-Degree and Post-college Earnings

**DOI:** 10.1007/s11162-019-09582-8

**Published:** 2019-10-26

**Authors:** Dirk Witteveen, Paul Attewell

**Affiliations:** 1 Nuffield College, University of Oxford, 1 New Road, Oxford, OX1 1NF UK; 2 The Graduate Center, City University of New York, 365 Fifth Avenue, New York, NY 10016 USA

**Keywords:** Delayed graduation, Time to degree, Work in college, Labor market outcomes, Bachelor’s degree, Earnings, Type of delay, Signalling, Human capital

## Abstract

Increasingly, undergraduates take more than 4 years to complete a baccalaureate, a situation widely perceived as a waste of time and money, for students, their families, and taxpayers. We first identify several phenomena that result in a longer time to degree and document the frequency of such delays. Then, using nationally representative data from the Baccalaureate & Beyond 1993–2003 surveys, we estimate the relationship between delayed time-to-degree and later employment and postcollege earnings, using negative binomial hurdle models. We find that delayed time-to-degree is not related to employment chances but is associated with lower post-college earnings: averaging 8–15%, depending on the length of delay. This average disadvantage is in line with signaling theory. The unique contribution of this study is its thorough analysis of different types of delay, as caused by stopping out and employment. Contrary to the popular assumption that delay is a waste of college resources or a student’s time, we find that delayed graduation in combination with working full-time during college has no negative relationship to post-college earnings. We discuss the time-investment trade-offs and the implications for the applicability of human capital theory to college graduation delays.

## Introduction

Conventionally, a bachelor’s degree in the US requires at least 120 credits, so a full-time student who takes 15 credits per semester can in principle complete a degree in eight semesters. Hence, a baccalaureate is popularly referred to as a “four-year degree.” Similarly, an associate degree involving 60 credits is known as a “two-year” degree. These are exemplary times to degree, however. National reporting systems reveal that many undergraduates take longer than the conventional 4 years to complete a bachelor’s, or longer than 2 years to complete an associate degree (National Center of Education Statistics (NCES) 2016a; Shapiro et al. 2016). Moreover, scholars report that the proportion of students who take longer to graduate has increased in recent decades, worsening the delay problem (Bound et al. 2012; Knight 2002, 2004).

A longer time-to-degree is widely perceived as a waste of a student’s time and money, as well as for society more broadly as it is a burden on colleges’ resources, and on taxpayers who support higher education (Complete College America 2014; DesJardins et al. 2002; Gilmore and Hoffman 1997). However, there is a dearth of research about whether students who take longer to graduate do suffer worse employment prospects or have lower post-college earnings than their classmates who graduated within the normative 4 years. Given the strong emphasis placed on time-to-degree as a college performance metric—and its consequences for educational policy—we view it as important to determine whether students who fail to graduate “on time” become disadvantaged in the labor market as a result.

This paper begins by documenting variation in time-to-degree in national data and discusses several social and institutional forces associated with delayed graduation. Next, it presents theories that suggest graduates who take a longer time to complete a degree should earn *less* after college, as well as counterarguments that this wage penalty is overstated because other forces will offset it. Subsequently, the paper analyzes nationally-representative longitudinal student data to answer two research questions: Are graduates who take longer to complete their degrees more or less likely to find employment after graduation? How do the post-college earnings of those who delay graduation compare to those who graduated ‘on time’?

This study’s novel contribution is a thorough analysis of the mechanisms of delay. Although the consequences of a delayed bachelor’s degree are relevant for our understanding of pathways in higher education, we are also interested in the *type* of delayed time-to-degree. Using data on labor market participation and enrollment history, conducted during graduates’ last year in college, we reveal some of the mechanisms—different components of human capital acquisition and conditions of a penalty through signaling—that explain an earnings delay-penalty after graduation, as well as the conditions under which these can be avoided. Several of our results have policy implications.

## Literature

### Dimensions of Time-to-Degree

On paper, virtually all bachelor’s degree programs require at least 120 credits to graduate. However, today’s baccalaureates accumulate on average 133.5 credits (Complete College America 2014). After a student has started college, several phenomena can lengthen time-to-degree. First, many undergraduates enroll on a part-time basis. In 2016, about 27% of undergraduates at 4-year colleges nationwide were part-timers.^1^ . By definition, part-timers enroll for a reduced number of course credits per semester, so it would be very difficult for them to complete a baccalaureate within 4 years. This part-time enrollment problem is sustained by federal regulations that treat enrolling for 12 credits per semester (rather than 15) as meeting the eligibility threshold for receiving full federal financial aid (such as a full Pell grant). Students who enroll for 12 credits per semester and who may think of themselves as attending full time cannot, as a matter of arithmetic, accumulate the 120 credits required for the degree within the normative 4-year period (Attewell and Monaghan 2016). Some students should be able to catch up on their credit attendance by summer course enrollment and thereby mitigate the total impact of part-time enrollment on delayed time to degree. However, current research has mainly focused on the positive impact of summer course-taking on retention and college completion (Attewell et al. 2012; Attewell and Jang 2013).

Second, about 40% of undergraduates entering 4-year colleges are required to take developmental or remedial courses in subjects such as mathematics or writing at the very beginning of the college career, usually because placement tests indicate that they have a skill deficiency (Boatman and Long 2018; Chen and Simone 2016; Radford and Horn 2012). Remedial courses typically do not carry college credit, so taking them fails to move the student towards the 120-credit finish-line for graduation. Consequently, despite the potential positive effect on completion chances, undergraduates who have to take remedial courses will not progress as fast towards graduation as they would in the absence of remedial coursework (Complete College America 2011).

Third, about 13% of entrants to 4-year colleges interrupt their enrollment during their first 3 years: they ‘stop out’ of college for some length of time (NCES 2016b: Table 2.1-b). There are diverse underlying reasons for this, such as job or family responsibilities and financial stresses (Broton and Goldrick-Rab 2016; St. John 2003). Despite the fact that a majority of stopouts do return to college at some point and subsequently graduate (Goldrick-Rab and Pfeffer 2009), their time-to-degree is necessarily lengthened by any time spent out of college.

Fourth, nowadays the majority of those who graduate with a baccalaureate have transferred institutions between their first enrollment in higher education and completing the degree: 56% in one recent cohort of graduates.^2^ For those who start at a 2-year or community college, a so-called vertical transfer is usually unavoidable if the goal is to earn a baccalaureate, but even among students who start at a 4-year college many transfer to another institution before graduating. Transfer can elongate time-to-degree if there is a time lag between leaving one college and enrolling in another. In addition to a time lag, when students transfer some find that their accumulated coursework from the first college is not fully credited towards the degree at the second institution (Simone 2014). This partial loss of credits means they have to take more coursework at the second institution than they would otherwise have done, had all their credits been accepted (Monaghan and Attewell 2015). As a result, transfers between colleges are expected to delay time-to-degree for bachelor’s students.

Fifth, some undergraduates delay before deciding on a major, while others start with one major but later change to another major. National Center for Education Statistics (2017) reports that 30% of students who have a declared a major at college entry change their major in the first 3 years of college. For some, this results in accumulating courses that “don’t count” towards the final major. Students in this situation may have to stay in higher education for longer until they have satisfied the course requirements for their new major. A variant on this situation occurs because the required coursework for certain majors exceeds 120 credits, sometimes because of professional accreditation requirements (Cataldi et al. 2011: 29). Other students ‘double-major’ during their college careers, which could take more time to complete than standard 4 years.

Having identified five ‘structural’ forces that almost inevitably increase time-to-degree—part-time enrollment, non-credit remedial coursework, transfers between institutions, stopping out, and changing or double majors—we now review evidence on how many undergraduates complete the baccalaureate within the traditional 4-year period, and conversely how many experience significant delays in graduation.

### Recent Evidence on Time-to-Degree and its Correlates

For several years, the National Center for Education Statistics (NCES) has undertaken longitudinal studies of recent college graduates, known as the Baccalaureate and Beyond (B&B) studies. Each B&B wave surveys a new nationally-representative sample of undergraduates who graduated from Title IV institutions with a baccalaureate degree in a particular year.^3^ Table 1 summarizes findings from three recent waves of the B&B, drawing from Bradburn et al. (2003, 2006) and from Cataldi et al. (2011).

**Table 1:** Time to degree by year of graduation for several national surveys of baccalaureates

Time to bachelor’s degree	1992/1993 (%)	1999/2000 (%)	2008/2009 (%)	2014/2015 (%)
48 months or less (4 years or less)	35	39	44	38
49 months thru 60 months (> 4 years to 5 years)	28	24	23	26
61 months thru 72 months (> 5 years to 6 years)	11	10	9	12
73 months thru 120 months (> 6 years to 10 years)	12	14	12	
More than 120 months (> 10 years)	14	14	12	
85 months thru 96 months (> 7 years to 8 years)				10
More than 96 months (> 8 years)				14

*Source* The first three columns report published figures from the National Center for Education Statistics’ Baccalaureate and Beyond Surveys, reported in Bradburn et al. (2003, 2006) and Cataldi et al. (2011). The rightmost column reports numbers from the National Student Clearinghouse system, reported in Shapiro et al. (2016: Table R-2)

For each wave of the B&B, fewer than half of those with bachelor’s degrees graduate within the normative 4-year period. Another 23 to 28% graduate within 5 years of college entry. For those, an extra semester or two is the extent of the delay. More striking is the substantial proportion who take much longer to graduate: for 2008/2009 graduates, 12% had taken between 6 and 10 years after starting college, and an additional 12% graduated more than 10 years after matriculating (Cataldi et al. 2011). A more recent cohort is profiled by Shapiro et al. (2016), using data from the National Student Clearinghouse (NSC). Their calculations—summarized in the rightmost column of Table 1—show that 24% of baccalaureates nationwide take over 7 years to graduate.

Both the B&B and the NSC studies document bivariate associations between time-to-degree on the one hand and certain institutional and student characteristics on the other. For example, time to the baccalaureate is much longer for students who start at community colleges (28% take over 10 years compared to 14% of all baccalaureates in the B&B). For students who graduate from for-profit colleges, the delay is even larger (45% take over 10 years). Time-to-degree is also longer on average for those who transfer between colleges, as well as for those who stop out of college for a time, and for those who delay between completing high school and first entering college (Cataldi et al. 2011; cf. Shapiro et al. 2016).

The recent B&B surveys also find that on average women complete their degrees faster than men, as has previously been found by Aina et al. (2011) and Löfren and Ohlsson (1999). These surveys further suggest that students with more highly educated parents are more likely to graduate on-time, although this effect does not hold in some studies with multivariate models (Lassibille and Navarro Gómez 2011). However, Bowen et al. (2009) do show that time-to-degree is substantially longer among students from low-income families.

In addition, B&B surveys report only very small differences in time-to-degree across racial and ethnic groups, even without controls for SES or for SAT scores. In terms of academic performance, students who enter with high SAT scores also typically graduate faster. Furthermore, according to the B&B studies, there are clear differences in time-to-degree between graduates who complete different majors: education, health, and engineering majors typically take longer to graduate (Cataldi et al. 2011).

In sum, using bivariate analyses prior research has shown that time-to-degree is associated with the type of institution attended, as well as with some demographic variables and the students’ route to the bachelor’s degree. However, these studies do not analyze the consequences of a longer time-to-degree in terms of labor market returns. A different research literature, reviewed next, considers post-college earnings and its relationship to delayed graduation.

### The Mincerian Approach

The dominant approach within economics for understanding education and earnings draws on Human Capital theory. For estimating the labor market consequences associated with a longer time-to-degree, it is imperative to review some of its theory’s principles. Human Capital theory rests on Becker’s (1962) assumption that individuals will achieve their educational level—i.e. a degree—if the net present value of the degree is positive at the time of enrollment. Any investment in education is perceived to be the result of a comparison between current and future monetary gains and the costs associated with enrollment.

 The notion of educational gains was further developed by Mincer (1974) in his influential book *Schooling, Experience and Earnings*. Mincer argued that the natural log of earnings ($\ln w$) is a function of the number of years of formal education (*s*) multiplied by a constant rate of return across time ($p_{s}$), plus a linear ($\beta_{0}$) and a quadratic ($\beta_{1}$) function of years of labor market experience (*x*), as shown in Eq. 1 for any individual (*i*).

1 $$\ln w\left( {s_{i} ,x_{i} } \right) = \alpha_{0i} + \rho_{si} s_{i} + \beta_{0i} x_{i} + \beta_{1i} x_{i}^{2} + \varepsilon_{i}$$

Thus, the more time spent in education *and* the more work experience, the higher the wage, on average. Workers with more education years benefit from a “compensating difference” in comparison to those with less education; they sacrificed accumulating work experience to spend time in school. Importantly, Eq. 1 does not make any assumption about credentials (having graduated or not). According to Mincer, the sheer time spent in formal education leads to higher earnings because it requires people to forego earnings. Furthermore, Mincer assumed the work experience would accrue *after* formal education. This implies a trade-off for individual labor market gains between the two value-adding components, namely years spent in school (*s*) and years spent in the labor market (*x*).

How would a Human Capital—i.e. Mincerian—approach predict the effect of delay time-to-degree? If a particular individual takes more time to reach a given degree than his or her fellow students do, then the delayed graduate’s potential years of post-college on-the-job experience are by definition reduced. Wages of a delayed graduate should therefore be lower. Moreover, some applications of Human Capital theory also expect delayed time-to-degree to be associated with lower wages in the short term because an interruption of schooling is likely to lead to Human Capital depreciation and obsolescence by itself (Fortin and Ragued 2017). Thus, either Mincerian reasoning suggests lower earnings for graduates with delayed time-to-degree, ceteris *paribus*.

### Complexities of the Mincerian Approach

Mincer’s model has been criticized by his fellow economists on several grounds—primarily for its neglect of tuition and other costs of education, for its treatment of the relationship between years of education and wages as linear rather than curvilinear, and for the “non-separability between schooling and earnings” (Heckman et al. 2003). Two additional lines of critique complicate the question of delay effects on earnings.

First, Mincer’s model, as well Becker’s (1964) original notion of “human capital,” was developed when most individuals first completed higher education and then entered the labor market, so that work experience began after graduation. In today’s academe in the United States, that pattern no longer holds: over half of the current generation of undergraduates works for pay while enrolled in college (Perna et al. 2007; Carnevale et al. 2015). This implies that work experience and course-taking (‘school years’) are often simultaneous rather than sequential and therefore need not be zero-sum. This is especially clear for the approximately 27% of undergraduates in 4-year colleges who attend college part-time. Attending part-time certainly increases time-to-degree, but it is unclear whether the part-timers’ pre-graduation on-the-job experience pays off as much in the post-college years in the labor market as the predicted loss of earnings from taking a longer time-to-degree.

Second, the Mincerian approach assumes that the *p*-coefficient in its Eq. (1) reflects an average (i.e. constant) return rate of education. However, in practice the expected earnings boost from years in education may vary throughout one’s career. In addition, as individual students gain more information about both the educational system and the labor market, they continuously revise their costs and benefits of the current college ‘investment’ (Aina et al. 2018). Such time-varying modifications—such as a job opportunity—could lead to a ‘rational’ decision to *delay* graduation. College students may (simultaneously) face immediate financial stress or (new) family responsibilities. As a result, many undergraduates “stop out”—do not enroll—for various periods between starting college and graduating, and many work for pay during those interludes. While stopping out lengthens time-to-degree (reducing the education payoff), it may at the same time be adding to a student’s job experience (*x*).

Third, although the theoretical framework of Becker and Mincer clearly notes the balance between costs and benefits, a more precise convention for predicting this ratio is to consider students’ decision to enroll or to work as decision-making ‘at the margins.’ (Toutkoushian and Paulsen 2016). This means that the balance between study and work is a function of the ‘internal rate of return’ on investment of both value-adding behaviors. More precisely, one prioritizes education when the marginal (or ‘internal’) rate of the *present* discounted value of college education (the current value of future benefits) exceeds that of the *present* discounted value of all direct costs (tuition) and indirect costs (foregone earnings). Not only can subtle shifts in the in the marginal rate of current earnings lead to more job seeking prior to graduation, it is also expected to yield a higher return in the long-term. Hence, working full-time during college can be an equally value-adding decision that can help offset some of the negative effects of delaying.

Finally, some economists have developed so-called random utility models to account for behavioral complexity in Human Capital models, including the non-linearity in the relationship between years of education and earnings (Manski 1989; Altonji 1993; Aina et al. 2018). Social scientists, however, have to turn to a more elaborate theoretical framework to predict the consequences of delayed time-to-degree. Their concern is the educational trajectory prior to graduation. Educational researchers have pointed to the (corresponding) perceptions of students regarding their educational and professional careers. For instance, in one national study of US undergraduates aged 24 or older, approximately two-thirds describe themselves as “employees who study” rather than as “students who work” (Berker et al. 2003).

### Recent Studies of Time-to-Degree

Recent studies have considered the impact of specific types of delay in their respective educational systems. Fortin and Ragued (2017) examine the relationship between stopping out and wages shortly after graduation, using Canadian data. As mentioned above, theory leads them to expect that a temporary interruption of schooling will “reduce real wage rates” due to human capital deprecation and obsolescence. They find, on the contrary, that undergraduates who stop out do *not* earn significantly less than counterparts who do not stop out. Probing further, they find that men who work full-time while stopping out actually earn more after graduation than their undergraduate counterparts who do not stop out. On average, women stopouts earn the same as women who do not stop out. However, the reasons for stopping out matter: students who stop out for health reasons, for example, do experience a post-graduation wage reduction.

Aina and Pastore (2012) examine delayed graduation and earnings, using data from Italy, and test two contrasting hypotheses. They initially hypothesize that Human Capital theory implies that spending a longer time in education should be associated with a higher post-college wage, because the extra education should result in additional skills. The contrary hypothesis, drawing on signaling theory, suggests that a graduate who delays and is older than the norm will suffer lower wages, since employers may read the delay as signifying lower skill or competence. Consequently, delayed graduates may have to take a job that typically requires less education than a degree, which they term “over-education.” Aina and Pastore’s (2012) empirical findings support the second (signaling) hypothesis: among Italian undergraduates, delayed graduation is associated with significantly lower wages and a much higher likelihood of over-education or over-qualification.

Recent research conducted with US data examines the wage benefits of attending college (Ma et al. 2016). Unlike the studies discussed previously, their predictive models include factors that condition short-term utility: the costs of attending college; both the direct costs of tuition, fees, and books and the indirect costs in foregone earnings while studying. They compare these costs to the post-college earnings of workers with different amounts of education. Their general finding is that a baccalaureate student who enters college at age 18 and pays the national average cost for college (without any grant aid) would recoup his or her college costs by age 34 through higher post-college wages. Thereafter the college graduate’s earnings would pull ahead of less-educated workers. Their main claim, therefore, is that “college pays” for graduates and (they find) pays even for students who only attend college for one year but do not graduate.

Ma et al. (2016) also consider scenarios that involve delayed graduation. They calculate, for example, that a baccalaureate holder who graduates in 5 years would “break even” and pay for their college costs by age 37, compared to an age 34 break-even point for someone who graduates in 4 years. This implies an earnings penalty from delayed graduation. While insightful, these calculations are based on several simplifying assumptions that include no earnings during the college years, no stopouts for work, and no part-time enrollment at lower tuition for the 5-year graduate. Those simplifying assumptions are unrealistic for many of today’s undergraduates.

## Analytical Strategy

How do employment chances and earnings from employment vary across graduates with different lengths of time spent in higher education? A classic Mincerian approach implies a significant overall earnings penalty for graduates with *any* delayed time-to-degree. However, time-to-degree studies from different countries suggest opposite and ambiguous effects. We will therefore undertake multivariate analyses of post-college wage data and time-to-degree for baccalaureate graduates in the United States, in order to determine whether these data indicate any association between time-to-degree and post-college earnings.

The unique contribution of our study is an analysis of several different *types* of delay and their distinct implications for post-college earnings. Given the Mincerian perspective on delayed graduation, one could still argue that, *if* there is a penalty for longer time to the bachelor’s degree, this might be mitigated by a wage benefit resulting from those extra years of work experience obtained while an undergraduate or during stopouts. We therefore ask whether longer pre-graduation stints in the labor market potentially counterbalance the initial delay penalty.

### Sample

One wave of the federal Baccalaureate and Beyond Study (B&B) drew a nationally-representative sample of persons who graduated with a bachelor’s degree in 1993/1994 and then re-surveyed the respondents a decade later, in 2003, when questions were asked about employment and earnings. The B&B data were obtained from the National Center for Education Statistics under a restricted license that requires us to round sample sizes reported below to the nearest ten, to ensure data confidentiality. Methodological details for the survey are provided by Wine et al. (2005).

The analyses are based on respondents who provided employment information 10 years after graduation and for whom full information on their time to degree could be derived based on their enrollment records—a precise measure detailed in number of months. We further restrict our B&B sample to bachelor’s graduates who were 21 years old or younger when they enrolled in college for the first time. Although adult students are increasingly more common in US higher education, they typically attain college with a different set of motivations and enrollment intensity (Bye et al. 2007). We further exclude the few survey respondents who were still enrolled in college 10 years after graduating with a baccalaureate, because including currently enrolled graduate students would add heterogeneity to our earnings models. Baccalaureates who had completed higher degrees by 2003 are included, however. Our overall goal is to understand the relationship between time to the baccalaureate and post-college labor market outcomes for typical college-age undergraduates, and our exclusions left us with a sample representative of that population. After exclusions, the sample size was 7130 persons.

Our research question requires a dataset that is able to provide detailed information on students’ higher education career. The B&B data contains such information, including attainment and achievement details, college major, type of 4-year institution or sector, and GPA, but also on students’ employment *during* college. The latter is an essential level of detail to explore mechanisms of types of delayed graduation. Furthermore, we make use of the 2003 observation because it is the most reliable source of the post-college labor market position. More recent datasets with shorter time frames, such as the B&B 2008–2012, suffer from high levels of current graduate school enrollment among bachelor’s degree graduates. This is problematic for building an accurate predictive model of labor market outcomes and earnings.

### Dependent variables

**Fig. 1:**
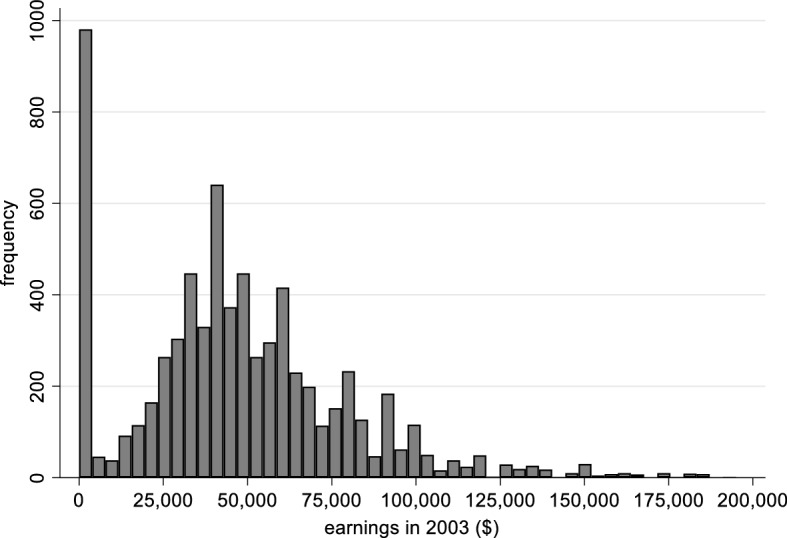
Distribution of Earnings (including 0-earner). *Notes* Baccalaureate and Beyond 1993–2003. Authors’ calculations of a subsample of students who entered higher education at age 21 or younger. N = 7130 (a rounded figure). Earnings above $200,000 are not included in this Fig. (49 cases)

Two variables function as dependent or outcome variables in our analyses: (a) whether a graduate was employed at the follow-up survey, roughly a decade after graduation, represented as a zero/one dummy variable; (b) the annual earnings of the graduate at follow-up (conditional on having positive earnings). Figure 1 shows the distribution of earnings for our sample.

### Operating Variable: Time-to-Degree

**Fig. 2:**
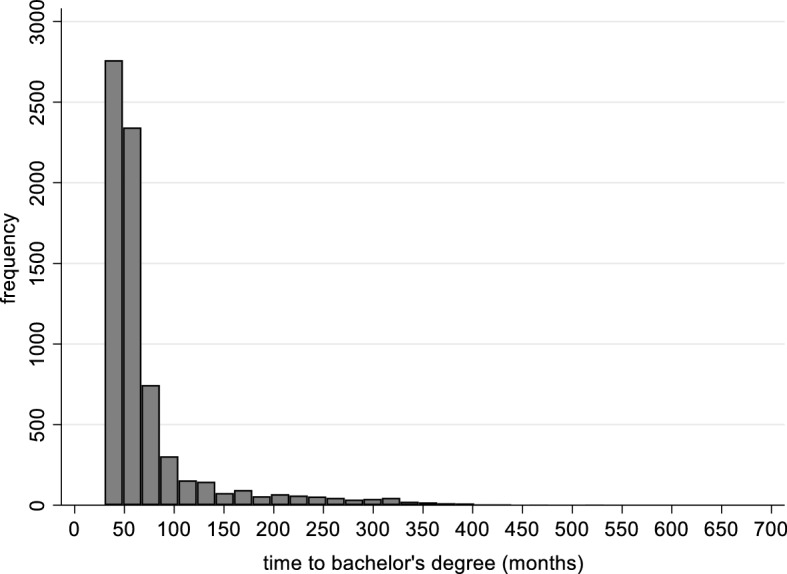
Distribution of Time to Bachelor’s Degree. *Notes* Baccalaureate and Beyond 1993–2003. Authors’ calculations of a subsample of students who entered higher education at age 21 or younger. N = 7130 (a rounded figure)

The B&B survey reported the date of entry into college and the graduation date for the baccalaureate, from which we calculated time-to-degree in months. However, this operating variable—time-to-degree—is very skewed (to the right), as documented in Fig. 2. We therefore operationalize time-to-degree using a categorical variable, a set of dummy variables intended to capture any non-linear effects. The categories are: (1) within 48 months (the reference category); (2) 49–60 months; (3) 61–72 months; (4) 73–120 months; (5) more than 120 months. This allows us to observe non-linearity in the delayed time-to-degree effects on earnings.

In additional analyses, we combined this time-to-degree measure with two other features of time spent *during* higher education: stopping out (at least once) and full-time employment (here defined as working more than 20 h—a significant burden for a college student). Those models evaluate any deviation from ‘no delay’—i.e. graduating within 48 months (1, the reference category) associated with: (2) a delay with stopouts during college, but no full-time work experience, (3) a delay with no full-time work experience, (4) a delay, with both stopouts and full-time work experience, and (5) a delay, with neither stopouts nor full-time work experience.

### Covariates

One study by Bound et al. (2012), using US data, did not find any empirical evidence correlating the variation of students’ characteristics with the elapsed time-to-degree. Nonetheless, we include students’ demographic information in our nested models to adjust for variation in labor market outcome (earnings) that are explained by these variables. The control variables used when predicting employment or earnings 10 years after graduation include gender, race/ethnicity, parental income, and parents’ highest educational attainment.

We also control for SAT/ACT score (strongly associated with selection into remedial coursework), a dummy variable for those who began college at a 2-year institution (who by definition require a transfer), a set of dummy variables representing the 9-level Carnegie classification for the type of 4-year institution that granted the baccalaureate, a dummy variable for graduating from a private institution, a 13-category college major variable (partially capturing longer curriculum), and college GPA. Appendix 1 provides descriptive statistics for all variables.

Modeling the relationship between time-to-degree and earnings is complicated by the fact that the dependent variable (earnings) is far from normally distributed, as is shown in Fig. 1. A substantial proportion of respondents have no or zero earnings at follow-up. For some this is because they are not employed; for a few they are employed but earn zero dollars per year. In statistical terms, this is “zero inflated” data. The right tail of the earnings variable is also much heavier than a normal or Gaussian distribution, even after excluding the very highest earners. Earnings is “over-dispersed.”

This means that, even were earnings logged, an ordinary least squares (OLS) regression predicting earnings from time-to-degree would be inappropriate statistically since the residuals would be far from normally distributed (Hilbe 2014: 17). We therefore instead employ a model recommended by Hilbe (2014: 184) for this kind of zero inflated and right-skewed dependent variable: a “two-part hurdle model.” This simultaneously estimates a logit model predicting who within the sample has zero earnings and also a negative binomial (NB) model predicting amount of earnings for those with non-zero earnings. The negative binomial distribution appropriately models both zero-inflated and over-dispersed count data (Hardin and Hilbe 2012; Hilbe 2011, 2014). The NB model is a generalization of a Poisson regression, but includes an extra parameter to model the over-dispersion in the dependent variable. The confidence intervals for the NB model are likely to be narrower compared to a traditional Poisson regression.

## Findings

### Non-linear Effects of Time-to-Degree

Table 2 reports descriptive statistics on time-to-degree and labor market status in the 1993–2003 B&B survey. Only 39% of the analysis sample earned the baccalaureate within 4 years; 11% took between 6 and 10 years and another 11% took longer than 10 years. Looking at the percent employed a decade after receipt of the baccalaureate, the average employment rate is 87.4%. There is no clear trend in employment by time-to-degree. However, the most-right columns of Table 2 suggest that there is a decline in mean dollar earnings as time-to-degree increases. These split earnings level by including non-earners and excluding non-earnings, respectively. For the latter, average earnings range from $61,791 for on-time graduates to $54,738 for those who took 10 or more years to graduate.

**Table 2:** Weighted dependent variables by time-to-degree

Time to bachelor’s degree	Graduates of 1993		Labor market status 2003		
	Frequency	Proportion	Percent employed (%)	Earnings (including 0-earners)	Earnings (among employed)
48 months or less (4 years or less)	2760	.387	87.4	$53,889	$61,791
49 months thru 60 months (> 4 years to 5 years)	1990	.279	87.8	$51,554	$58,876
61 months thru 72 months (> 5 years to 6 years)	770	.108	87.1	$49,783	$57,201
73 months thru 120 months (> 6 years to 10 years)	790	.110	88.0	$48,965	$55,714
More than 120 months (> 10 years)	820	.115	86.0	$46,860	$54,738
Total	7130	1.000	87.4	$51,316	$58,880

Baccalaureate and Beyond 1993–2003. Authors’ calculations of a subsample of students who entered higher education at age 21 or younger. N = 7130 (a rounded figure)

**Fig. 3:**
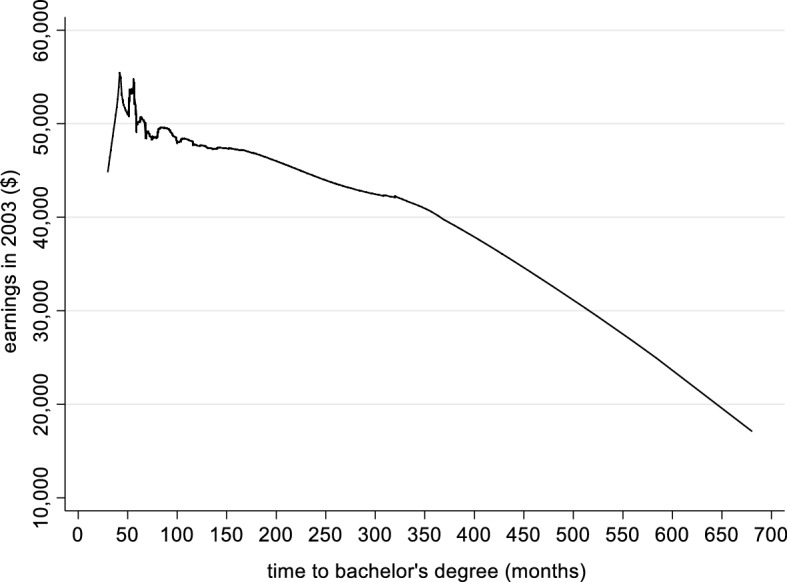
Locally Weighted Scatterplot (LOWESS) of Time-to-Degree and Earnings. *Notes* Baccalaureate and Beyond 1993–2003. Authors’ calculations of a subsample of students who entered higher education at age 21 or younger. N = 7130 (a rounded figure). Smoothing bandwidth: .8

A visual representation of the relationship is provided in Fig. 3, which shows a smoothed (Lowess) curve showing the bivariate relationship between time-to-degree and earnings 10 years after college. This shows a steady drop in earnings immediately after the normative 48 months of college, implying an earnings disadvantage related to delayed time-to-degree. We will test whether this drop can be associated with earnings dispersion in a multivariate context in the hurdle models that follow.

Table 3 summarizes three “nested” two-part hurdle models. The left-hand column reports Model 1, a baseline model with only time-to-degree predicting employment and earnings, with no controls. Model 2 adds controls for pre-college demographic variables such as gender, race/ethnicity, and parental education and parental earnings. Model 3 adds additional control variables describing college major and type of institution attended. (The full list of covariates appears at the bottom of Table 3 and the full models with coefficients for all variables including controls are reported in Appendix 2.)

**Table 3:** Coefficients predicting earnings in 2003: a negative binomial-logit hurdle regression

	Model 1 baseline (no controls)		Model 2 (+ pre-college demographics)		Model 3 (+ pre-college demographics & college factors)	
	β	s.e.	β	s.e.	β	s.e.
Logit (predicting having 0 earnings)^a^						
49 months thru 60 months (> 4 years to 5 years)	.025	.086	− .074	.091	− .098	.098
61 months thru 72 months (> 5 years to 6 years)	.133	.124	− .079	.137	− .138	.144
73 months thru 120 months (> 6 years to 10 years)	.129	.123	.040	.168	− .058	.176
More than 120 months (> 10 years)	− .049	.115	.148	.186	.083	.203
Negative binomial (predicting earnings among > 0)^a^						
49 months thru 60 months (> 4 years to 5 years)	− .084***	.018	− .081***	.018	− .069***	.019
61 months thru 72 months (> 5 years to 6 years)	− .089***	.025	− .085***	.026	− .061**	.026
73 months thru 120 months (> 6 years to 10 years)	− .114***	.015	− .065***	.033	− .042	.033
More than 120 months (> 10 years)	− .151***	.018	− .074***	.037	− .049	.039
Summary statistics						
N	7130		7130		7130	
Wald Chi^2^	2.60		323.16		419.14	
Prob. > Chi^2^	.626		.000		.000	
LL	− 75,243		− 74,680		− 74,391	
AIC	21.110		20.958		20.887	

Baccalaureate and Beyond 1993–2003. Authors’ calculations of a subsample of students who entered higher education at age 21 or younger. N = 7130 (a rounded figure). Pre-college demographics: gender, race/ethnicity, parental education, and parental income. College factors: SAT score, started in a 2-year institution, private/public college, Carnegie classification, major, and college GPA. See Appendix 2 for the coefficients of the control variables

^a^Reference category is 48 months or less (4 years or less). Significance levels

*p < .05, **p < .01, and ***p < .001 (two-sided)

Each hurdle model contains three panels, stacked vertically. The top panel reports the logit-part of the model that predicts who has zero earnings from the set of dummy variables representing time-to-degree. In none of the three models is time-to-degree significantly associated with zero earnings. We infer that delayed time-to-degree does not negatively affect one’s chances of post-college employment about 10 years after graduation.

The middle panel reports coefficients for the negative binomial-part of models, predicting the amount of earnings among those with non-zero earnings. Looking at Model 1 (with no controls) each of the dummy variables representing longer time-to-degree is statistically significant and negative: compared to the reference category—the baccalaureate within 4 years—a longer time-to-degree is associated with lower earnings 10 years later. As the model predicts the log of the expected counts (of dollar earnings) as a function of the predictors, a coefficient reflects the difference in logs of dollars for a one-unit increase in the predictor. This may be interpreted as approximately the percentage difference in earnings between the reference category and the active category. Thus, those who graduate between 49 months and 60 months of entering college earn on average 8.4% less than on-time graduates, conditional on having positive earnings, while those who take over 10 years to graduate earn on average 15.1% less than on-time graduates (in Model 1).

Additional covariates for pre-college demographics, including family background, as added in Model 2, do not change the picture very much. The logit model coefficients remain non-significant, suggesting that time-to-degree does not affect whether one is employed or has non-zero earnings. However, the coefficients for time-to-degree in the negative binomial model are all negative and statistically significant, suggesting that delayed time-to-degree is associated with lower average earnings 10 years after graduation, regardless of demographic background.

Nonetheless, after adjusting for demographics, the coefficients for a time-to-degree of 6–10 years and greater than 10 years are reduced in magnitude, compared to the baseline model. This suggests that differences in family background explain some of the lower earnings associated with longer time-to-degree; however, the coefficients for longer time-to-degree are still significantly different from zero. Also noteworthy is the fact that baseline-model coefficients for 49–60 months and 61–72 months are barely affected by adding demographic controls in Model 2. A further sensitivity analysis (Appendix 3) indicates that parental income and parental education are among the most prominent control variables that reduce the negative effect size of a longer time degree on post-college earnings.

Finally, Model 3 adds to the previous demographic controls a set of covariates that describe college factors: the type of institution attended (Carnegie classification); whether the undergraduate began at a 2-year or 4-year college (vertical transfer); whether the student attended a private or public institution; several measures of student performance (SAT score, college GPA); and college major at graduation. Adjusting for these various college factors does make the coefficients for the two longest times to degree statistically non-significant, although the magnitude of the coefficients does not change much compared to Model 2. We interpret this as meaning that the association between long time-to-degree and post-college earnings may partly be a reflection of the kind of the institution and program attended or the academic performance of long time-to-degree undergraduates. We also note in Model 3 that, after all these controls, the coefficients for 49–60 months and 61–72 months remain statistically significant compared to ‘on-time’ graduates. As documented in Appendix 3, students’ performance indicators (SAT score and college GPA), and to a lesser extent college type and college major, have the largest impact on the non-significant effects of the longest time to degree categories.

Subsequently, since existing literature suggests that women, racial- and ethnic minorities, and lower-income (or class) students are more likely to take longer to graduate, we tested whether these demographics can also be associated with higher levels of earnings penalty, depending on the length of time-to-degree. However, none of the tested interactions between the time-to-degree predictor and these demographics yielded significant estimates with exception of one. After controlling for all covariates, women who take more than 120 months to graduate (10 years) experience, on average, a disproportionally higher earnings penalty. This interaction term is substantial (.119, CI .024, .213) and statistically significant (p = .014), yet the confidence interval is relatively large. Despite the high variance, we interpret this as an additional earnings disadvantage of delayed graduation for women.

Together, these analyses suggest an earnings penalty for *any* delayed college graduate, observed 10 years after graduation. This deficit is non-linear; a longer time-to-degree increases the size of the earnings disadvantage, yet our models suggest that this growing effect size is partly associated with individual demographic features, including family background, performance, and college contexts. However, we also observe heterogeneous effects; those graduates who took much longer to graduate (more than 6 years) have attended different institutions and programs than those who take delay with less than 2 years.

### Pre-graduation Trade-Offs

Although a wide range of factors may lead to heterogeneity in the impact of delayed time-to-degree, we are most concerned with pre-graduation trade-offs experienced by today’s college graduates. Most specifically, we are interested in the impact of an increasingly common pattern in higher education—delay because of stopping out—on post-college earnings, as well as the human capital implications of full-time work experience before college graduation.

**Fig. 4:**
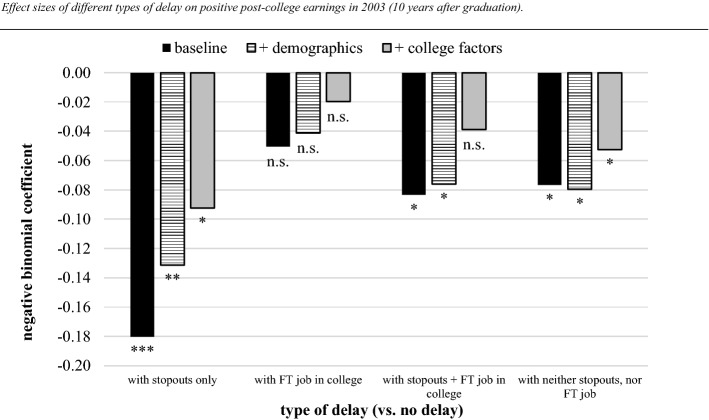
Effect sizes of different types of delay on positive post-college earnings in 2003 (10 years after graduation). *Notes* Baccalaureate and Beyond 1993–2003. Authors’ calculations of a subsample of students who entered higher education at age 21 or younger. N = 6190 (a rounded figure). The coefficients reported are from a negative binomial regression on non-zero (positive) earnings. Pre-college demographics: gender, race/ethnicity, parental education, and parental income. College factors: SAT score, started in a 2-year institution, private/public college, Carnegie classification, major, and college GPA. Significance levels: *p < .05, **p < .01, and ***p < .001 (two-sided)

Using the same covariates list as in our initial models (Appendix 1), we estimate the relative impact of different types of delay and its combinations. Holding no delay (i.e. on-time graduation within 4 years) as the reference categories, Fig. 4 displays the effect size and significance levels of four different combinations of stopping out and working full-time. These results have been estimated from a negative binomial regression as part of the comprehensive hurdle model. Similar to our initial estimates of time-to-degree, we found no effect of types of delay on the likelihood of having employment 10 years after graduation.

However, leftmost in Fig. 4, we observe that a delayed time-to-degree while having stopped out is associated with a significant earnings deficit. While these graduates may have held side-jobs, they did not work full-time before graduation. For this type of delay, the baseline model suggests a loss in logs of dollar earnings of .18 (about 20%). After adjusting for demographics and college-specific features, such as the type of institution and major, the overall impact of delaying while stopping out is still substantial, about a .092 logs reduction (− 9.7%) in post-college earnings. About 12% of delayed graduates are in this group.

In contrast, students who take longer than expected to graduate, but at the same time hold a full-time job before graduation, do not experience an earnings deficit (as indicated by the non-significant second group of effect size bars). This type of trade-off between time to degree and working for pay while in college is most common among delayed graduates; about 40% of the B&B sample are in this group, conditional on taking longer than 4 years to the BA. It is important to note that although they worked during college, these graduates did *not* stop out during their entire 4-year college career.

What happens when stopping out is combined with full-time work experience? About 16% of delayed graduates fall into this group who stop out in order to (temporarily) be employed in a full-time job. As seen the third group of bars, both the baseline- and demographics-adjusted models indicate an earnings penalty of about 7.9%. However, after controlling for institutional and performance features the significance of this deficit disappears and its effect is reduced to .039 (logs). We interpret this negative effect of stopping out as being cancelled out by pre-graduation full-time employment, yet only if taken place in specific institutions and programs.

The rightmost group of bars summarize those students who delayed time-to-degree *in college*. We call this an ‘in college delay’ because they neither have a history of stopping out (thereby extending their time-to-degree), nor holding a full-time job before graduation (to potentially counterbalance the negative delay-effect). In other words, based on observing no hiatus in their enrollment, nor a full-time job that could have taken up too much time, we may infer that these students delayed for other reasons than competing responsibilities related to work or taking time off. About 32% of delayed graduates fall in category. For this group, we do find a significant earnings deficit 10 years after graduation of about 6 to 8%, which is robust to controls for demographics and college-specific factors. Potentially relevant interactions of type of delay and demographics (e.g. gender) are not statistically significant (not shown).

### Length of Delayed Full-Time Working Graduates

Since we observed that full-time work experience before degree completion is not significantly associated with an earnings penalty in the post-college career, we wanted to see if this holds for *any* length of delay (i.e. time-to-degree). We ran interactions between the previously used categories of time-to-degree and a dummy for full-time work before college graduation. Table 4 presents the negative binomial regressions on positive earnings, nested in the same model specifications as before.

**Table 4:** Interaction coefficients predicting earnings in 2003 (10 years after graduation): a negative binomial regression

	Model 1 baseline (no controls)		Model 2 (+ pre-college demographics)		Model 3 (+ pre-college demographics & college factors)	
	β	s.e.	β	s.e.	β	s.e.
Negative binomial (predicting earnings among > 0)^a^						
1 thru 12 months delay * no full-time job	− .027	.047	− .054	.039	− .029	.037
13 thru 24 months delay * no full-time job	− .085	.053	− .112**	.050	− .101*	.048
25 thru 72 months delay * no full-time job	− .189**	.049	− .164**	.052	− .133**	.052
More than 72 months delay * no full-time job	− .231**	.048	− .142**	.051	− .103*	.053
No delay * with full-time job	.031	.036	− .001	.032	− .003	.031
1 thru 12 months delay * with full-time job	− .042	.033	− .063	.031	− .048	.032
13 thru 24 months delay * with full-time job	− .048	.048	− .075	.045	− .040	.044
25 thru 72 months delay * with full-time job	− .039	.058	− .012	.059	.001	.054
More than 72 months delay * with full-time job	− .059	.038	− .045	.051	− .029	.049
Summary statistics						
N	6190		6190		6190	

Baccalaureate and Beyond 1993–2003. Authors’ calculations of a subsample of students who entered higher education at age 21 or younger. N = 6190 (a rounded figure). The coefficients reported are from a negative binomial regression on non-zero (positive) earnings 10 years after graduation. Pre-college demographics: gender, race/ethnicity, parental education, and parental income. College factors: SAT score, started in a 2-year institution, private/public college, Carnegie classification, major, and college GPA

^a^Reference category is no delay (48 months or less) with no full-time job during college

Significance levels: *p < .05, **p < .01, and ***p < .001 (two-sided)

When reading the top-panel of Table 4 from left to right—baseline to full model—we observe a statistically-significant interaction reflecting an earnings penalty for delayed time-to-degree with no full-time work experience, with the exception of a short delay of two semesters (12 months) or less. These coefficients reflect the effect sizes (up to − .133) vis-à-vis the reference category: having no delayed path to the BA graduation and no full-time job experience. These interaction effects confirm our earlier finding of a substantial earnings penalty of delay for students who neither stop out nor hold a full-time job during college.

In contrast, none of the coefficients in the bottom panel indicate disadvantages in terms of earnings for those who held a full-time job before graduation. This reflects a remarkable null finding: for those graduates who accumulated full-time work experience during college, the length of time spent between initial enrollment and graduation creates no labor market disadvantage at all.

## Discussion and Conclusion

Traditional Human Capital theory remains ambiguous on how delayed time-to-degree effects post-college in modern labor market outcomes. Nowadays, US higher education pathways increasingly involve interruptions (stopouts) and simultaneous work experience acquisition (pre-graduation paid full-time work). It is unclear from economic theory whether these negative and positive factors cancel each other out. Nationally-representative surveys suggest a steady increase in recent years of non-normative (4-year) college career lengths, both in terms of short delays and very long delays (more than 6 years). Despite the popular assumption that a delay between college entry and college graduation is problematic—primarily wasting resources for educational institutions and individual students—there has been little prior research on this topic.

Our analyses of nationally representative data on baccalaureate graduates document that students whose graduation is delayed are just as likely to be employed for pay a decade after graduation, but on average we observe that delayed graduates earn significantly less—between 8 and 15% less—than their classmates who graduate on time (within 4 years of starting college). Thus, a longer time-to-degree is associated with a progressively increasing earnings penalty. This finding is in line with signaling theory applied in other countries (e.g. Aina and Pastore 2012). It also in general agreement with Ma et al.’s (2016) analysis of ‘break-even’ points (ages) in US education, which implied an income disadvantage for non-on-time college graduates.

We do not assume that the association between delay and earnings deficits is causal. We find that the earnings penalty associated with delayed graduation is associated to some extent with the family background of delayed students, and the types of institution they attend and students’ major and GPA. However, even after such factors are statistically controlled for, there remains a significant association between earnings and delayed time-to-degree: a 7–8% earnings deficit. We also find it noteworthy that the earnings penalty associated with delayed time-to-degree is not just evident for graduates with long delays; on the contrary, the earnings gap is sizeable even for students who graduate five or 6 years after college entry—a year or two of delay. There is no evidence for the earnings penalty operating differently for graduates of different parental and racial-ethnic backgrounds. However, women who take more than 10 years to graduate experience a slightly higher earnings deficit than men. This gender difference has not been reported in earlier research on inequalities in education. (See Buchmann et al. (2008) for a comprehensive review).

We then further distinguished the association between time-to-degree and earnings by the *types* of delay that are increasingly common in college trajectories: working full-time before graduation and stopping out. Both work-enrollment ‘strategies’ are direct or indirect components of the Mincer equation. Our analyses reveal three important mechanisms. First, delayed time-to-degree is not associated with a labor market disadvantage when it occurs in the context of full-time employment prior to graduation, regardless of whether one also stopped out (in order to work more). In other words, the negative impact of delayed time-to-degree—as predicted by one part of the Mincer equation—is completely cancelled out by the other human capital-increasing component: work experience. Here, we only selected full-time work—work that is assumed to be most relevant for acquiring relevant (job) skills. In further robustness analyses, we noticed that delayed time to the baccalaureate degree can be of *any* length, but is not associated with lower post-college earnings if full-time employment occurred before graduation.

However, when delayed graduation occurs in the absence of a full-time job, stopping out is associated with an earnings penalty of about 10% after adjusting for demographics and college factors. Moreover, those students delay graduation but have an ‘in college delay’—they neither stopped out nor held a full-time job—experience a substantial earnings disadvantage 10 years after graduation. Stopping out may result from a multitude of factors, including family responsibilities or illness. These mechanisms are however beyond the scope of this study.

To our knowledge, the comprehensive perspective on delay (due to work during college) has not been studied in the US context. The fact that the negative impact of delay on mid-career earnings disappears for (full-time) working students suggests that relevant human capital can be acquired not only through its general form (education) or specific (firm) human capital, but also through work experience before entering the first job. This suggests a human capital investment ‘trade-off’, for college students, between increasing their human capital and prioritizing *current* income by working (more) in college, versus working less in college, only gaining human capital through education, graduating on-time (not suffer from the signaling penalty) and increasing *future* income. Our findings suggest that for mid-career incomes the former route is indistinguishable from the latter. This conclusion contracts the popular assumption that delay is a waste of college resources or a student’s time (or investment).

However, in addition to the overall association between delayed time to degree and lower mid-career earnings, the reduction in earnings is more prevalent when delay is not combined with full-time work before graduation. This finding is in line with signaling theory—employers may perceive delayed graduates with an ‘in college delay’ as having no good reason for their slower study pace and therefore consider ten less desirable hires. Future research should therefore focus on the potential individual-level and institutional-level factors that contribute to this least desirable type of delayed graduation.

What can colleges do to reduce graduates’ delay penalty? One policy recommendation is focused on reducing the delay itself. While some factors that affect time-to-degree are partly a matter of student choices—stopping out, changing major, part-time attendance—colleges may also contribute to some extent to delayed degrees—by allowing some majors to require well over 120 credits for graduation, or by tight restrictions for awarding full credit to some transfer students, for example. Knowing that there are earnings consequences for delayed degrees should spur college administrators to make adjustments where possible to facilitate students’ timely progress towards graduation and to identify and remove hurdles that slow students down. For instance, students who are at risk of delaying because of family commitments or busy work schedules may benefit from flexibility in their course schedules, such as choice of evening classes, online classes, or particular summer classes. This type of course taking will also help already delaying students to keep up or reduce their time-to-degree.

Furthermore, because time-to-degree’ complexity in relation to stopouts and work matters for earnings after graduation, colleges should pay attention to the particular context or circumstances of delayed students. Our study indicates that a uniform policy encouraging *all* students to graduate on time is unlikely to improve post-college outcomes for several subgroups of students. Such a ‘one-size-fits-all’ approach would ignore students who combine full-time work with coursework for whom, as we have shown, delay is on average not consequential for future earnings. This means that a more customized approach to working students would be preferred over simply discouraging (delaying) working students away from their current work life (which is often a necessity to make ends meet). At the same time, however, our study shows that delay in combination with stopouts (i.e. no high number of work hours) and a lower level of work experience does indeed lead to higher earnings penalties. Thus, the take-away for college counseling is that one should aim to evaluate the contexts of delaying students early on. Encouragement to maintain a high level of credit taking could be beneficial for some students but detrimental for others, and vice versa.

## Appendix 1

See Table 5.

**Table 5:** Weighted descriptive statistics

	Proportion	Mean
Dependent variables		
Employed in 2003	.874	
Earnings in 2003 (including 0-earners)		$51,316
Earnings in 2003 (among employed)		$58,880
Operating variable		
Time to bachelor’s degree (months)		78.03
Control variables		
Gender		
Male	.455	
Female	.545	
Race/ethnicity		
White	.845	
Black	.058	
Hispanic	.049	
Asian	.044	
Native/other	.004	
Parental income (among dependents)		$65,757
Highest degree parents		
Less than high school	.030	
High school/GED	.197	
Vocational school	.085	
Some college	.075	
Associate’s degree	.067	
Bachelor’s degree	.260	
Post-graduate degree	.285	
Age at college entry		17.99
SAT/ACT score		
1st quartile (bottom)	.164	
2nd quartile	.193	
3rd quartile	.231	
4th quartile (top)	.220	
Did not take SAT or ACT	.192	
Started in a 2-year institution	.163	
Carnegie classification		
Research I	.239	
Research II	.085	
Doctorate-granting I	.069	
Doctorate-granting II	.070	
Comprehensive I	.330	
Comprehensive II	.026	
Liberal arts I	.055	
Liberal arts II	.093	
Other	.034	
Private institution	.332	
Major		
Humanities	.102	
Social/behavioral science	.149	
Life sciences	.071	
Physical sciences	.017	
Mathematics	.014	
Computer/information science	.024	
Engineering	.067	
Education	.125	
Business/management	.247	
Health	.056	
Vocational/technical	.021	
Other technical/professional	.094	
Other/non-coded	.013	
College GPA		3.03

Baccalaureate and Beyond 1993–2003. Authors’ calculations of a subsample of students who entered higher education at age 21 or younger. N = 7130 (a rounded figure). Missing cells on the control variables are imputed 20 times

## Appendix 2

See Table 6.

**Table 6:** Coefficients of the control variables predicting earnings in 2003: a negative binomial-logit hurdle regression

	Model 2 (pre-college demographics)				Model 3 (pre-college demographics & college factors)			
	β logit	s.e.	β negative binomial	s.e.	β logit	s.e.	β negative binomial	s.e.
Gender (female)	1.432***	.125	− .353***	.020	1.375***	.130	− .299	.020
Race/ethnicity^a^								
Black	− 1.663*	.768	.246	.264	− 1.725*	.804	.199	.219
Hispanic	− .006	.271	.065	.051	− .040	.281	.019	.049
Asian	− .450	.240	− .022	.035	− .339	.237	.002	.036
Native/other	− .067	.199	.013	.039	.001	.200	.003	.037
Parental income^b^								
Dependent, 1st quartile	.169	.162	− .112**	.044	.152	.164	− .104	.040
Dependent, 2nd quartile	.209	.150	− .110**	.035	.265	.165	− .098	.034
Dependent, 3rd quartile	− .085	.160	− .121**	.035	− .059	.162	− .113	.034
Independent, 1st quartile	.220	.210	− .178***	.044	.223	.212	− .134	.041
Independent, 2nd quartile	.144	.228	− .181***	.043	.171	.233	− .151	.040
Independent, 3rd quartile	.399	.255	− .157**	.051	.475	.250	− .135	.046
Independent, 4th quartile	.612*	.304	.005	.057	.699*	.301	.009	.053
Parental education^c^								
Less than high school	.001	.255	− .114*	.049	.022	.253	− .067	.047
High school/GED	− .407**	.152	− .076*	.034	− .382*	.154	− .061	.030
Vocational school	− .388*	.191	− .134**	.039	− .386*	.194	− .099	.037
Some college	− .232	.174	− .041	.038	− .232	.176	− .024	.036
Associate’s degree	− .096	.223	− .053	.038	− .084	.223	− .034	.037
Post-graduate degree	.046	.117	− .038	.029	− .028	.117	− .031	.028
SAT/ACT score^d^								
1st quartile (bottom)					.107	.186	− .009	.035
2nd quartile					− .070	.151	− .065	.028
4th quartile (top)					.217	.141	− .016	.027
Did not take SAT or ACT					− .109	.150	.041	.032
Started in 2-year institution					− .329*	.149	− .038	.025
Carnegie classification^e^								
Research I					.247	.140	.083	.027
Research II					.371*	.161	− .010	.032
Doctorate-granting I					.425*	.196	.081	.040
Doctorate-granting II					− .008	.175	.066	.054
Comprehensive II					.179	.271	− .127	.048
Liberal arts I					.171	.206	.021	.045
Liberal arts II					.049	.185	− .172	.033
Other					.502	.307	.004	.053
Private institution					.204	.114	.066	.023
Major^f^								
Humanities					.222	.172	− .123	.040
Life sciences					− .368	.195	− .050	.039
Physical sciences					− .297	.343	− .011	.058
Mathematics					− .139	.324	− .093	.059
Computer/information sc.					− .307	.428	.180	.058
Engineering					− .247	.262	.110	.038
Education					.175	.156	− .219	.032
Business/management					− .367*	.177	.084	.035
Health					− .530*	.244	.074	.040
Vocational/technical					− 1.538**	.527	− .014	.061
Other tech./professional					− .435*	.192	− .009	.040
Other/non-coded					.161	.299	− .009	.076
College GPA^g^								
1st quartile (bottom)					− .023	.143	− .046	.024
2nd quartile					.405	.132	− .022	.027
3rd quartile					.593	.129	.032	.028

Baccalaureate and Beyond 1993–2003. Authors’ calculations of a subsample of students who entered higher education at age 21 or younger. N = 7130 (a rounded figure)

Reference categories: ^a^white, ^b^dependent, 4th quartile (top), ^c^bachelor’s degree, ^d^3rd quartile, ^e^comprehensive I, ^f^social/behavioral science, ^g^4th quartile

Significance levels: *p < .05, **p < .01, and ***p < .001 (two-sided)

## Appendix 3

See Tables 7 and 8.

**Table 7:** Change in model 1 negative binomial earnings coefficients when stepwise adding model 2 predictors

	**(1)** 48 months or less vs.:			
	**(2)** 49–60 months	**(3)** 61–72 months	**(4)** 73–120 months	**(5) >** 120 months
Model 1 coefficients	− .084***	− .089***	− .114***	− .151***
Added one at a time				
+ Gender	− .113***	− .142***	− .164***	− .118***
+ Race/ethnicity	− .083***	− .087**	− .110***	− .144***
+ Parental income	− .056**	− .033	− .014	− .107**
+ Parental education	− .071***	− .072**	− .091***	− .113***

**Table 8:** Change in model 2 negative binomial earnings coefficients when stepwise adding model 3 predictors

	**(1)** 48 months or less vs.:			
	**(2)** 49–60 months	**(3)** 61–72 months	**(4)** 73–120 months	**(5)** **>** 120 months
Model 2 coefficients	− .081***	− .085***	− .065***	− .074***
Added one at a time				
+ SAT score	− .063***	− .058*	− .039	− .058
+ Started 2-year institution	− .083***	− .084**	− .065*	− .079*
+ Private/public college	− .084***	− .086**	− .064	− .075*
+ Carnegie classification	− .091***	− .094***	− .071*	− .063
+ Major	− .095***	− .090***	− .071*	− .071
+ College GPA	− .075***	− .073**	− .052	− .071

## Footnotes

1 Part-time is defined by the federal IPEDS system as enrolling in fewer than 12 credit hours per semester. The 27% figure is the authors’ calculation using data from Table 4 in the National Student Clearinghouse report “Current Term Enrollments Spring 2016.” Retrieved December 10, 2018. (https://nscresearchcenter.org/currenttermenrollmentestimate-spring2016/)

2 Calculated using NCES’ EasyStats online analysis tool using data from the 2008/2009 wave of the B&B study.

3 Title IV institutions refer to colleges and universities that are classified under Title IV of the Higher Education Act of 1965 as qualified for the federal student financial aid system. In practice federal statistical agencies use Title IV eligibility as a way to define which colleges and universities are included in their statistical coverage.

## References

[CR1] Aina C, Baici E, Casalone G (2011). Academic drop-out and the great recession. Education Economics.

[CR2] Aina, C., Baici, E., Casalone, G., & Pastore, F. (2018). The economics of university dropouts and delayed graduation: A survey. Discussion paper no. 11421. Institute for the Study of Labor Economics.

[CR3] Aina, C., & Pastore, F. (2012). Delayed graduation and overeducation: A test of the human capital model versus the screening hypothesis (discussion paper no. 6413). Institute for the Study of Labor Economics.

[CR4] Altonji JG (1993). The Demand for and return to education when education outcomes are uncertain. Economics of Education Review.

[CR5] Attewell P, Heil S, Reisel L (2012). What is academic momentum? And does it matter?. Educational Evaluation and Policy Analysis.

[CR6] Attewell P, Monaghan D (2016). How many credits should an undergraduate take?. Research in Higher Education.

[CR7] Becker GS (1964). Human capital: A theoretical and empirical analysis, with special reference to education.

[CR8] Berker, A., Horn, L. J., & Carroll, C. D. (2003). Work first, study second: adult undergraduates who combine employment and postsecondary enrollment (report no. 2003-167). National Center for Education Statistics. Retrieved 10 Dec, 2018, from https://nces.ed.gov/pubs2003/2003167.pdf.

[CR9] Boatman A, Long BT (2018). Does remediation work for all students? How the effects of postsecondary remedial and developmental courses vary by level of academic preparation. Educational Evaluation and Policy Analysis.

[CR10] Bound J, Lovenheim MF, Turner S (2012). Increasing time to Baccalaureate degree in the United States. Education Finance and Policy.

[CR11] Bowen WG, Chingos MM, McPherson MS (2009). Crossing the finish line: Completing college at America’s public universities.

[CR12] Bradburn, E. M., Berger, R., Li, X., Peter, K., Rooney, K., & Griffith, J. (2003). A descriptive summary of 1999–2000 bachelor’s degree recipients 1 year later, with an analysis of time-to-degree (report no. 2003-165). National Center for Education Statistics. Retrieved 10 Dec, 2018 from https://nces.ed.gov/pubs2003/2003165.pdf.

[CR13] Bradburn, E. M., Nevill, S., Cataldi, E. F., & Perry, K. (2006). Where are they now? A description of 1992–93 bachelor’s degree recipients 10 years later (report no. 2007-159). National Center for Education Statistics. Retrieved Dec, May 10, 2018, from https://nces.ed.gov/pubs2007/2007159a.pdf.

[CR14] Broton K, Goldrick-Rab S (2016). The dark side of college (un)affordability Food and housing insecurity in higher education. Change: The Magazine of Higher Learning.

[CR15] Buchmann C, DiPrete TA, McDaniel A (2008). Gender inequalities in education. Annual Review of Sociology.

[CR16] Bye D, Pushkar D, Conway M (2007). Motivation, interest, and positive affect in traditional and nontraditional undergraduate students. Adult Education Quarterly.

[CR17] Carnevale, A. P., Smith, N., Melton, M., & Price, E. W. (2015). Learning while earning: The new normal. Georgetown University Center on Education and the Workforce. Retrieved December 10, 2018, from https://cew.georgetown.edu/wp-content/uploads/Working-Learners-Report.pdf.

[CR18] Cataldi, E. F., Green, C., Henke, R., Lew, T., Woo, J., Shepherd, B., & Siegel, P. (2011). 2008–09 Baccalaureate and Beyond Longitudinal Study (BB:08/09): First Look.” Report no. 2011-236. National Center for Education Statistics. Retrieved Dec, May 10, 2018, from https://nces.ed.gov/pubs2011/2011236.pdf.

[CR19] Chen, X., & Simone, S. A. (2016). Remedial coursetaking at U.S. public 2- and 4-year institutions: Scope, experiences, and outcomes (report no. 2016-405). National Center for Education Statistics. Retrieved Dec, May 10, 2018, from https://nces.ed.gov/pubs2016/2016405.pdf.

[CR20] Complete College America. (2011). Time is the enemy: The surprising truth about why today’s college students aren’t graduating… and what needs to change. Complete College America. Retrieved December 10, 2018, from https://www.luminafoundation.org/files/resources/time-is-the-enemy.pdf.

[CR21] Complete College America. (2014). Four-year myth: Make college more affordable. Restore the promise of graduating on time. Complete College America. Retrieved December 10, 2018, from https://completecollege.org/wp-content/uploads/2017/05/4-Year-Myth.pdf.

[CR22] DesJardins SL, Ahlburg DA, McCall BP (2002). A temporal investigation of factors related to timely degree completion. Journal of Higher Education.

[CR23] Fortin B, Ragued S (2017). Does temporary interruption in postsecondary education induce a wage penalty? Evidence from Canada. Economics of Education Review.

[CR24] Gilmore GM, Hoffman PH (1997). The graduation efficiency index: Validity and use as an accountability and research measure. Research in Higher Education.

[CR25] Goldrick-Rab S, Pfeffer FT (2009). Beyond access: Explaining socioeconomic differences in college transfer. Sociology of Education.

[CR26] Hardin JW, Hilbe JM (2012). Generalized linear models and extensions.

[CR27] Heckman, J. J., Lochner, L. L., & Todd, P. E. (2003). Fifty years of mincer earnings regressions (working paper no. 9732). National Bureau of Economic Research. Retrieved December 10, 2018, from http://www.nber.org/papers/w9732.pdf.

[CR28] Hilbe JM (2011). Negative binomial regression.

[CR29] Hilbe JM (2014). Modeling count data.

[CR30] Knight, W. E. (2002). Toward a comprehensive model of influences upon time to bachelor’s degree attainment (report no. 85). Association for Institutional Research. Retrieved December 10, 2018, from http://airweb3.org/airpubs/85.pdf.

[CR31] Knight, W.E. (2004). Time to bachelor’s degree attainment: An application of descriptive, bivariate, and multiple regression techniques (report no. ED504365). Association for Institutional Research. Retrieved Dec, May 10, 2018, from https://archive.org/details/ERIC_ED504365.

[CR32] Lassibille G, Navarro Gómez ML (2011). How long does it take to earn a higher education degree in Spain?. Research in Higher Education.

[CR33] Löfren C, Ohlsson H (1999). What determines when undergraduates complete their thesis? Evidence from two economics departments. Economics of Education Review.

[CR34] Ma, J., Pender, M., & Welch, M. (2016). Education pays 2016: The benefits of higher education for individuals and society. The College Board. Retrieved December 10, 2018, from https://trends.collegeboard.org/sites/default/files/education-pays-2016-full-report.pdf.

[CR35] Manski CF (1989). Schooling as experimentation: A reappraisal of the post-secondary dropout phenomenon. Economics of Education Review.

[CR36] Mincer J (1974). Schooling, experience, and earnings.

[CR37] Monaghan D, Attewell P (2015). The community college route to the bachelor’s degree. Education Evaluation and Policy Analysis.

[CR38] National Center for Education Statistics. (2016a). Persistence and attainment among postsecondary subbaccalaureate students (report no. 2016-083). National Center for Education Statistics. Retrieved December 10, 2018, from https://nces.ed.gov/pubs2016/2016083.pdf.

[CR39] National Center for Education Statistics. (2016b). First-time postsecondary students in 2011-12: three-year withdrawal, stopout, and transfer rates.” Report no. 2016-139. National Center for Education Statistics. Retrieved December 10, 2018. https://nces.ed.gov/pubs2016/2016139.pdf.

[CR40] National Center for Education Statistics. (2017). Beginning college students who change their majors within 3 years of enrollment (report no. 2018-434). National Center for Education Statistics. Retrieved December 10, 2018, from https://nces.ed.gov/pubsearch/pubsinfo.asp?pubid=2018434.

[CR41] Perna LW, Cooper MA, Li C, StJohn EP (2007). Improving educational opportunities for students who work. Readings on equal education: Confronting educational inequality: Reframing, building, understanding, and making change.

[CR42] Radford, A. W., & Horn, L. J. (2012). Web table: An overview of classes taken and credits earned by beginning postsecondary students (report no. 2013-151REV). National Center for Education Statistics. Retrieved December 10, 2018, from https://nces.ed.gov/pubs2013/2013151rev.pdf.

[CR43] Shapiro, D., Dundar, A., Wakhungu, P. K., Yuan, X., Nathan, A., & Hwang, Y. (2016). Time-to-degree: A national view of the time enrolled and elapsed for associate and bachelor’s degree earners (signature report no. 11). National Student Clearinghouse Research Center.

[CR44] Simone, S. A. (2014). Transferability of postsecondary credit following student transfer or coenrollment (statistical analysis report 2014-163). National Center for Education Statistics. Retrieved December 10, 2018. https://eric.ed.gov/?id=ED546652.

[CR45] St. John EP (2003). Refinancing the college dream: Access, equal opportunity, and justice for taxpayers.

[CR46] Toutkoushian RT, Paulsen MB (2016). Economics of higher education. Background, concepts, and applications.

[CR47] Wine, J. S., Cominole, M. B., Wheeless, S., Dudley, K., Franklin, J., & Perry, K. (2005). 1993/03 Baccalaureate and Beyond longitudinal study (methodology report no. 2006–166). National Center for Education Statistics. Retrieved December 10, 2018, from https://nces.ed.gov/pubs2006/2006166.pdf.

